# Feasibility and efficacy of bypassing the right ventricle and pulmonary circulation to treat right ventricular failure: an experimental study

**DOI:** 10.1186/1749-8090-7-15

**Published:** 2012-02-06

**Authors:** Jan Spillner, Christian Stoppe, Nima Hatam, Andrea Amerini, Ares Menon, Christoph Nix, Ulrich Steinseifer, Yousef Abusabha, Hanna Giessen, Rüdiger Autschbach, Marcus Haushofer

**Affiliations:** 1 Department for Cardiothoracic- and Vascular Surgery, University Hospital RWTH Aachen, Pauwelsstr. 30, D-52074 Aachen; 2 ABIOMED Europe GmbH; Neuenhoferweg 3, D-52074 Aachen; 3 Applied Medical Engineering (CVE), Helmholtz Institute Aachen, Pauwelsstr. 20, D-52074 Aachen; 4 Department of Anesthesiology, University Hospital RWTH Aachen, Pauwelsstr. 30, D-52074 Aachen

**Keywords:** Right ventricular failure, surgical treatment, assist device, pulmonary circulation, bypass, gas exchange, perfusion route

## Abstract

**Background:**

Right ventricular failure (RVF) and -support is associated with poor results. We aimed for a new approach of right - sided assistance bypassing the right ventricle and pulmonary circulation in order to better decompress the right ventricle and optimize left ventricular filling.

**Methods:**

From a microaxial pump (Abiomed), a low resistance oxygenator (Maquet and Novalung) and two cannulas (28 and 27 Fr) a system was set up and evaluated in an ovine model (n = 7). Connection with the heart was the right and left atrium. One hour the system was operated without RVF and turned of again. Then a RVF was induced and the course with the system running was evaluated. Complete hemodynamic monitoring was performed as well as echocardiography, flow measurement and blood gas analysis.

**Results:**

The overall performance of the system was reliable. Without RVF no relevant changes of hemodynamics occurred; blood gases were supra normal. In RVF a cardiogenic shock developed (MAP 35 ± 13 mmHg, CO 1,1 ± 0,7 l/min). Immediately after starting the system the circulation normalized (significant increase of MAP to 85 ± 13 mmHg, of CO to 4,5 ± 1,9). Echocardiography also revealed right ventricular recovery. After stopping the system, RVF returned.

**Conclusions:**

Bypassing the right ventricle and pulmonary circulation with an oxygenating assist device, which may offer the advantages of enhanced right ventricular decompression and augmented left atrial filling, is feasible and effective in the treatment of acute RVF. Long time experiments are needed.

## Background

Right ventricular failure is an often deleterious condition and associated with a high mortality [1,2]. The pathophysiology of RVF is complex, reflecting the challenges in treatment of RVF [3]. Surgical options to treat RVF are very limited and besides transplantation the only proven surgical option is a right ventricular exclusion with a "fontanisation" of blood circulation [4,5]. Conventional implantation of assist devices in RVF shows poor long-term results [6], what in our point of view is due to the fact that major mechanisms of RVF are not eliminated despite mechanical circulatory assistance. An important mechanism, which leads to low cardiac output in RVF, is the reduced trans-pulmonary blood flow with reduced left atrial-, and consecutively left ventricular filling. The major determinant of right ventricular performance is right ventricular afterload. In previous studies on RVF we focused on a passive (and oxygenated) decompression of the pulmonary circulation into the left atrium and could demonstrate significant hemodynamic improvement [7,8]. Taking all this into account we hypothesized that an optimal assist system to treat RVF should take over the stroke work of the right ventricle on the one hand, and should both reduce right ventricular afterload and augment left ventricular filling by bypassing the pulmonary circulation into the left atrium on the other hand. Based on the fact, that this system bypasses the lung a gas exchange within the system is essential because otherwise severe hypoxemia would result. We designed such a system with forthcoming potential for an ambulatory application and named it "Right-Left Assist Device" (R-L AD). This device was evaluated in an open chest animal model of acute RVF assessing feasibility, efficacy to treat RVF and possible adverse effects.

## Methods

### I Development and characterisation of the system

#### I a) Configuration and set up

**Figure 1:**
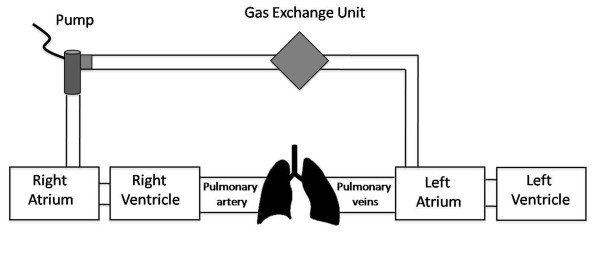
**Arrangement scheme of the R-L AD; note, that the main principle of the system is bypassing the right ventricle and the pulmonary circulation**. Delivery of oxygenated blood to the left atrium results in a controlled oxygenated right to left shunt.

Our aim was to create an extracorporeal active pump system to deliver blood from the right atrium directly to the left atrium. To prevent hypoxia an artificial gas exchange system had to be integrated. Therefore the systems' main components consisted of a blood pump, an oxygenator and cannulas connecting the system with the heart. [Figure 1: Arrangement scheme of the R-L AD]

#### I b) Pump

The Impella^® ^Elect 600 microaxial pump (Abiomed Europe GmbH, Aachen, Germany) was used to generate active blood flow. This miniaturized rotary blood pump has a diameter of 12 mm, a length of 40 mm and a weight of 6 g. The inner volume of the pump is only 1,5 mL and the inner artificial and blood contacting surface is 15,9 cm^2^. At a maximal speed of 33000 revolutions per minute (rpm), a flow of up to 6 L/min can be generated. All control parameters are displayed continuously on the external driving console [9,10].

#### I c) Gas-exchange unit

In order to utilise maximum performance of the pump, "low resistance" oxygenators with the smallest possible resistance were used. Initially we chose the Quadrox^® ^(Maquet, GmbH & Co KG Rastatt, Germany), which includes an effective heat exchanger. This oxygenator bears a 1,8 m^2 ^polymethylpentene diffusion membrane and can be run with a blood flow rate from 0.5 to 7 L/min. The Quadrox^® ^system was used in 2 animals. After the first 2 experiments we switched to oxygenator to the Novalung^® ^- iLA membrane oxygenator (Novalung GmbH, Hechingen, Germany) due to the relevant degree of unhandiness of the Quadrox^® ^system (bigger size and weight, need for a gas blender). The Novalung^®^'s diffusion membrane of polymethylpentene has a surface of 1,3 m^2 ^and is housed in a rather small rigid 15 × 15 cm polyethlen box. It is intended for a blood flow of 0,5 to 4,5 L/min.

#### I d) Cannulas, connections and complete system

**Figure 2:**
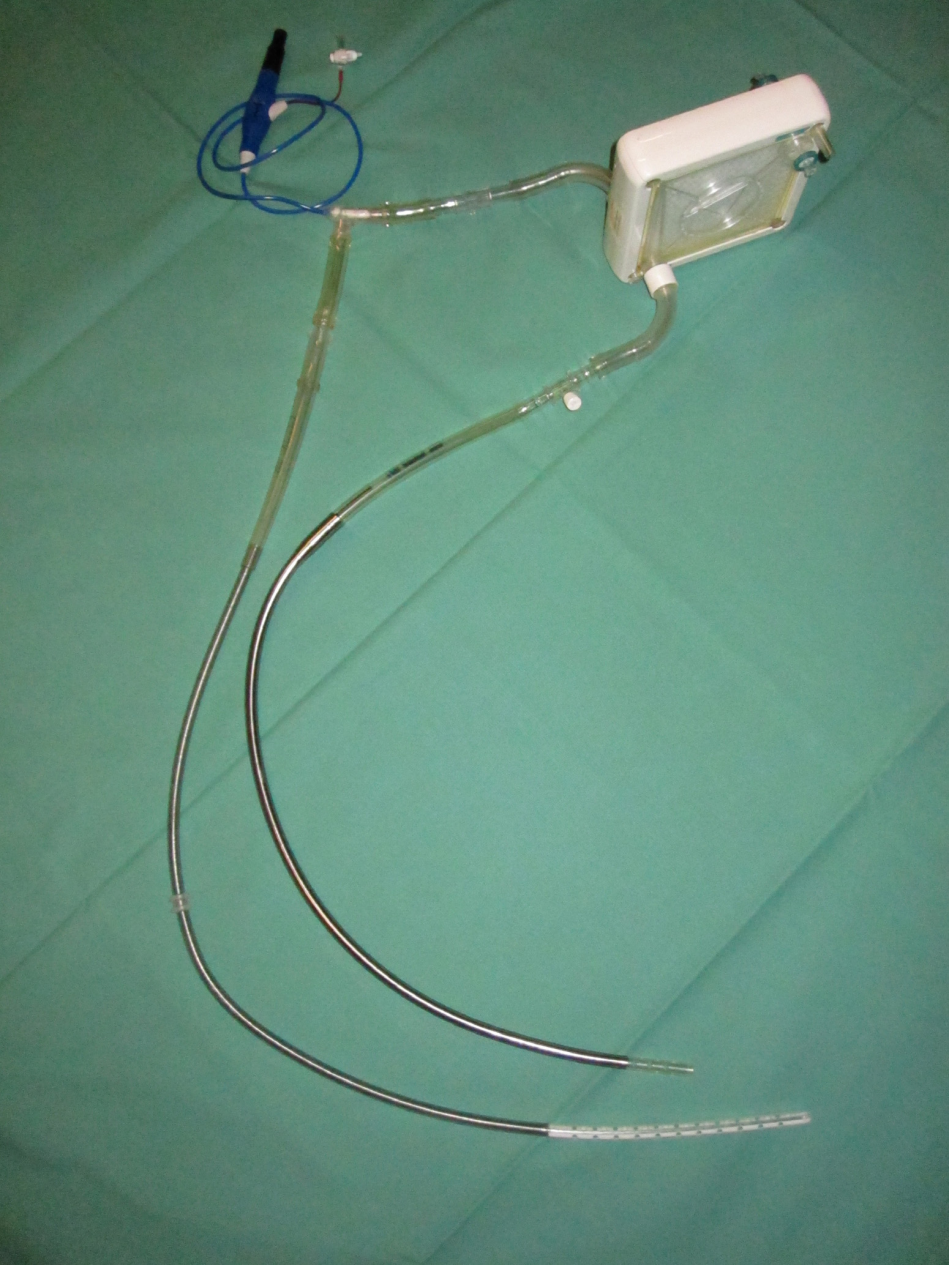
**Photograph of the system ("R-L AD"); it consists of the pump (top left), the oxygenator (top right) and the cannulas (bottom)**.

A 28 Fr cannula (Edwards Lifesciences^®^, USA) with a length of 88 cm was used for evacuating the blood out of the right atrium. This cannula was connected via a 3/8 (inch) flexible tube (Raumedic^®^; Helmbrechts, Germany) of 5 cm length to the microaxial pump. On the other side of the pump's connector another 5 cm long 3/8 tube connected to the gas exchange unit. Following the gas exchange unit another 5 cm of flexible tube is connected to a 27 Fr cannula ("Bio-Medicus" Femoral Venous Cannula, Medtronic^®^, USA) of 70 cm length delivering the blood into the left atrium. [Figure 2: Photograph of the system ("R-L AD")]

### II Experimental evaluation in the animal model

After having completed the setup of our "Right-Left Assist Device", we investigated it in seven animals. In principle the experiments should characterize the system with and without RVF.

#### II a) Animals and anaesthesia

Seven female Rhoensheeps of 65 (+/- 4) kg were used in this study. The animal research protocol was approved by the local authorities (Lanuv in Recklinghausen; Ref No.: 8.87-51.04.20.09.307). Animals were treated according to the "Guide for the care and use of Laboratory animals" published by the US National Institutes of Health (National Institutes of Health publication no. 85-23, revised 1996). After premedication, anesthesia was maintained by continuous intravenous application of pentobarbital and fentanyl Animals were intubated and ventilated initially with 100% oxygen. Tidal volumes were adjusted to 10 ml/kg in order to maintain a pCO_2 _of 4,65 - 5,32 kPa according to the blood gas analyses. Oxygen concentration was maintained at 40%. Single shot antibiotic prophylaxis was administered (Cefuroxime 1,5 g) before skin incision. Fluid (Ringers lactate) was given at a rate of 20 ml/kg/h. The temperature of the animal was maintained with a heating blanket and an infrared lamp. During the operation of the oxygenators the Quadrox^® ^system was run with a flow rate of 4 L/min and a FiO_2 _of 0.6 via a gas blender. The Novalung^® ^was run with 4 L/min of oxygen during the whole experiment.

#### II b) Instrumentation and surgical preparations

All animals received a central venous line (Quad-Lumen Indwelling Catheter, Arrow^®^, USA) via the left internal jugular vein. Both of them were dissected surgically. A Swan-Ganz catheter (7.5-F, Model VS 1721; Ohmeda, UK) was inserted into the proximal pulmonary artery from the left - sided jugular vein. Another balloon catheter ("Berman" Angiographic Catheter 6 Fr 60 cm; Arrow^®^, USA) was advanced into the right ventricle from the right jugular vein to determine right ventricular pressures. The left - sided groin was surgically dissected with exposition of the femoral vessels. Using Seldingers technique a femoral arterial line (18 G, Vygon, Germany) was placed into the left femoral artery under direct vision.

A small left thoracotomy in the fourth intercostal space was performed. After incising the pericardium, the left atrium was exposed and pulmonary artery was dissected. A catheter (18 G, Vygon^®^, Germany) was inserted into the left atrium and secured with a suture. All catheters were connected to fluid - coupled pressure transducers. An ultrasonic flow probe (24 mm Transsonic^®^; USA) was placed around the proximal descending aorta. After complete heparinization a purse-string suture was placed on the free lateral wall of the left atrium. The 27 Fr outflow cannula was introduced into the left atrium after incision of the wall, clamped and suture secured. Thereafter the 28 Fr inflow cannula was positioned in the right atrium via the right jugular vein under echocardigraphic control and also clamped. As a last step the whole system was carefully deaired and connected with the cannulas. The pump speed was adjusted to approximately 29.000 rpm resulting in a flow of 4,2 l/min, controlled by an ultrasonic flow probe (Transsonic^®^, USA) around the 3/8 inch tube.

#### II c) Right ventricular failure model

Banding of the very distal main pulmonary artery was performed to achieve RVF. We used a custom-made apparatus to reduce the diameter of the pulmonary artery very slowly by a screwing mechanism. The banding was persistent. RVF following pulmonary artery banding was defined as a profound decrease in systemic blood pressure (MAP < 2/3 of baseline) and depressed cardiac output (CO < 2/3 of baseline). Additionally right ventricular function was judged by visual inspection.

#### II d) Echocardiography

Transthoracic echocardiography was performed in all experiments using the Vivid E 9 System with the M5S Transducer (GE Vingmed, Germany) at 2.5 MHz. In a four chamber equivalent view, the right ventricle was imaged in standard two-dimensional mode for online estimation of systolic function by global morphology, right ventricular diameter (RVd) measured at the tricuspid annulus and tricuspid annular plane systolic excursion (TAPSE) in M-mode. TAPSE ≥ 18 mm was regarded as normal. In addition right ventricular failure was estimated by the immediate occurrence of ventricular septal shift to the left during the acute increase in right ventricular afterload. A cine loop of at least four consecutive cardiac cycles was stored for off-line analysis by Echopac software (GE Vingmed, Germany).

#### II e) Experimental Protocol

The animal experiment should prove the principal feasibility of our new approach and allow a characterization of the system during different circulatory conditions. Therefore measurements were taken at predetermined times. These measurements ("data acquisition" (DA)) included complete hemodynamic monitoring, flow measurement and blood gas analysis. After having finished all surgical preparations and having the system connected (but clamped) a "baseline I" measurement was performed. In a first step the running R-L AD was evaluated during a period of normal circulation. This was followed by a "baseline II" with an again turned off and clamped R-L AD system. Thereafter RVF was induced and characterized. This was followed by "treatment of RVF" by switching on the R-L AD during persistent pulmonary artery banding. To exclude meantime changes in right ventricular function or any other circulatory adaptations, the R-L AD was switched off again to characterize the degree of the "recurrent RVF (Re-RVF)". The echocardiographic investigations (E) were performed during unaffected circulation, during RVF and during the "treatment of RVF".

At the end animals were euthanized with an overdose of pentobarbital and fentanyl. A macroscopic autopsy of the entire carcass was carried out with focus on the heart, lungs and kidneys. [Additional file 1: Experimental protocol]

#### II f) Data acquisition and statistical analysis

Data were collected using the Datex AS-3 Compact Monitor (Datex Engstrom, USA) and digitally recorded during the study. Flow measurements were also collected and digitally recorded during the study. Data are expressed as means +/- SD. Statistical analyses were performed with version 18 of SPSS (SPSS, USA). A Student's 2 tailed paired t test was used. All statistical analyses were performed to a level of significance of 0,05.

## Results

### a) General results

**Figure 3:**
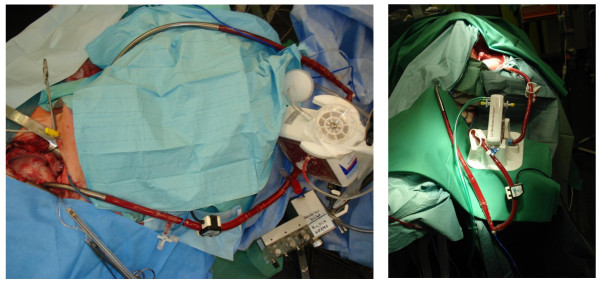
**Operative view of the R-L AD; Left: with the Quadrox^®^, Right: with the Novalung^®^**.

All animals survived the whole experiment until recurrence of RVF with the R-LAD turned off again. During the next 30 min after re-initiating RVF 4 of the 6 animals died due to severe cardiogenic shock. The remaining 2 animals also suffered severe cardiogenic shock and therefore had to be euthanised. Surgical preparations were uneventful. The placement of the cannula from the jugular vein into the right atrium was uncomplicated under echocardiographic guidance. Insertion of the left atrial cannula via the lateral thoracotomy also proved to be easy and safe. Only very little contamination of air was detectable during the insertion of the cannula into the left atrium. Cardiopulmonary bypass was not necessary at any time of the operation. Neither catecholamines had to be administered in any experiment nor relevant arrhythmia occured. During the whole experiment urine output maintained at normal levels except the longer lasting recurrent RVF. The R-L AD was easily manageable: operation of the device was completely without dysfunction. It run stable without any relevant alterations. The aimed flow of 4,2 l/min could be delivered at pump speeds of 29.000 rpm despite the different conditions during the course of the experiment. Gas exchange worked very well with both types of oxygenators. [Figure 3: Operative view of the R-L AD's] The visible "collapse" of the right ventricle indicated effective decompression during system operation without RVF. In RVF tachycardia with severe changes in the electrocardiogramm (new right bundle branch block with ST-segment depression and inversed T-wave) occurred with an obviously severe overdistension of the right ventricle confirmed by echocardiography (*E 2*). When the R-L AD was switched on, the right ventricle recovered nearly immediately. "Eyeballing" again showed a collapsing aspect of the right ventricle resembling its morphology without RVF and pump-support as shown above. The electrocardiogramm normalized with a delay of some minutes. With the R-L AD turned off again, RVF reoccurred with the same overdistended aspect of the right ventricle and changes in the electrocardiogram.

### b) Hemodynamics and flows

During the experiments all hemodynamic parameters and flows were digitally recorded. An overview of the most relevant data is given in Additional file 2, Table S2. [Additional file 2: Relevant data from the experiments]

#### "R-L AD"

In this section the effect of the system in an unaffected circulatory status was studied. The overall hemodynamics stayed completely stable. No relevant alteration of the cardiac output was detectable with similar arterial pressures compared to the baseline measurements. The only alterations were a significant decrease of the CVP from 8 to 3-4 mmHg with an inversely proportional increase of the LAP from 9 to 20-23 mmHg. Amazingly right ventricular filling pressures decreased only slightly (ns). The pO2 increased significantly from 13 to 29-30 kPa, also leading to an elevated mixed venous oxygen saturation.

#### "RVF"

Right ventricular failure demonstrated severe with a significant decrease of CO from 3.9 to 1.1 L/min and concordant of the MAP from 75 to 35 mm Hg. CVP rose up to 19 whereas the LAP decreased to 3-5 mmHg. Especially the latter is one of the most important features of RVF with decreased left sided filling (serial ventricular interdependence). The right ventricular filling pressures initially increased, but with the severity of RVF decreased again. The mixed venous oxygen saturation declined significantly from 66 to 39% indicating a severe cardiogenic shock.

#### "Treatment of RVF"

all signs of cardiogenic shock disappeared immediately by turning on the R-L AD. CO significantly recovered to 5.1 (+/- 1,5) l/min at most, the MAP, after an initial overshoot up to 85 mmHg, normalized around 78 (+/- 8) mmHg, which is significant in comparison to "RVF". The mixed venous saturation also normalized to 70 (+/- 4.4) % with an arterial pO2 around 30 kPa. The major reason for the overall recovery (with an interrupted vicious circle of the RVF) is augmented left atrial filling in conjunction with right sided decompression: the CVP declined significantly from around 18 to 4 mm Hg, but more important the LAP rose from around 4 to 22 mm Hg. This hemodynamic alteration is clearly caused by the R-L AD because blood is pumped from the right atrium to the left atrium.

#### "Re-RVF"

**Figure 4:**
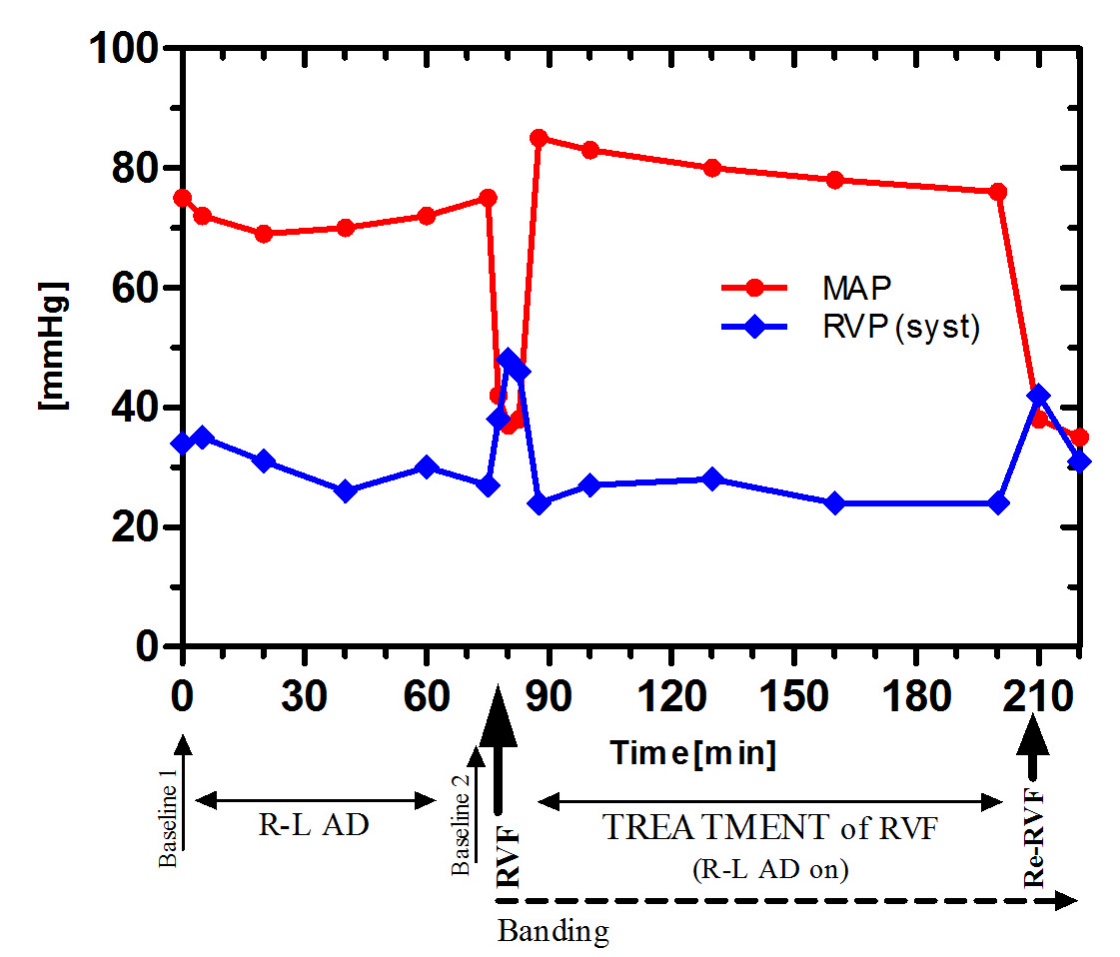
**Diagram of the time course of MAP and RVP**. Note the nearly unchanged pressures despite the running R-L AD between baselines I and II. In RVF the MAP normalizes when the R-L AD is switched on (after initial overshoot).

**Figure 5:**
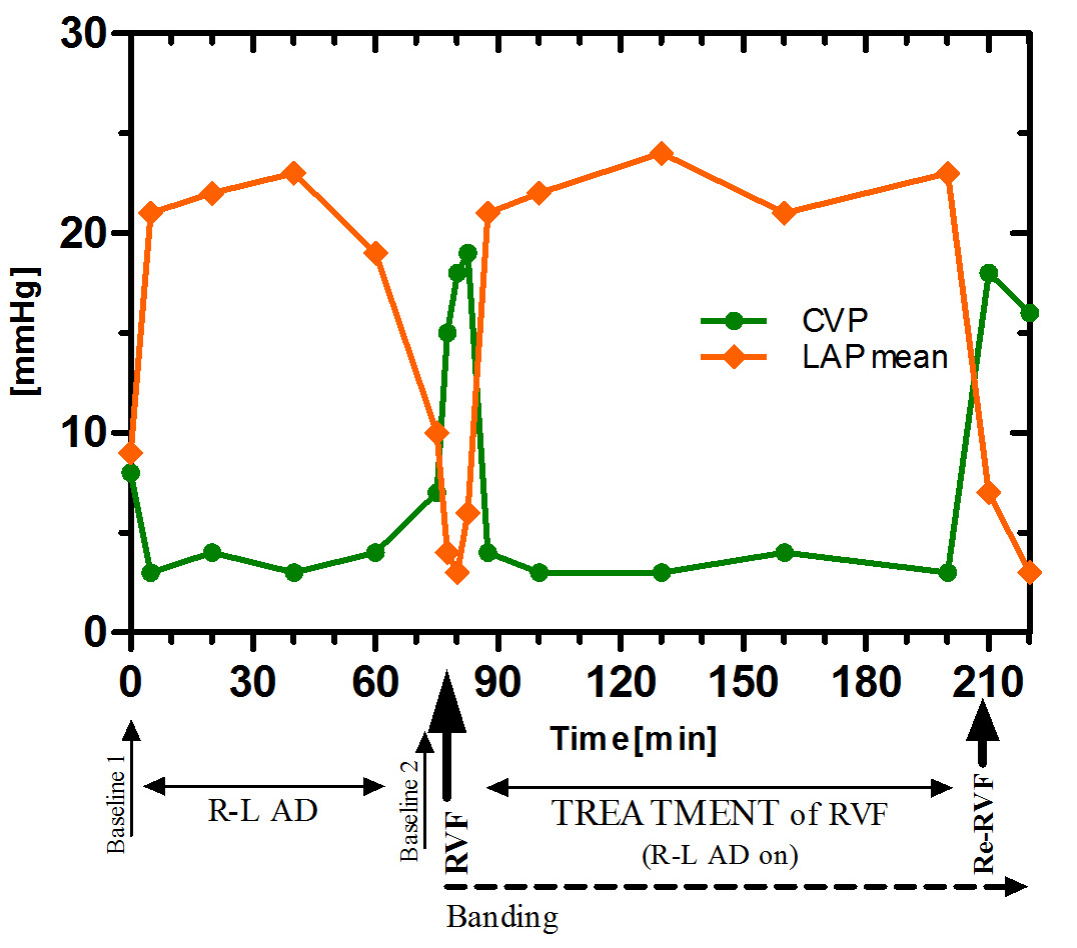
**Diagram of the time course of CVP and LAP during the experiment demonstrating the efficacy of bypassing the right ventricle and pulmonary circulation by the R-L AD (decompression of right heart with augmentation of left sided filling)**.

**Figure 6:**
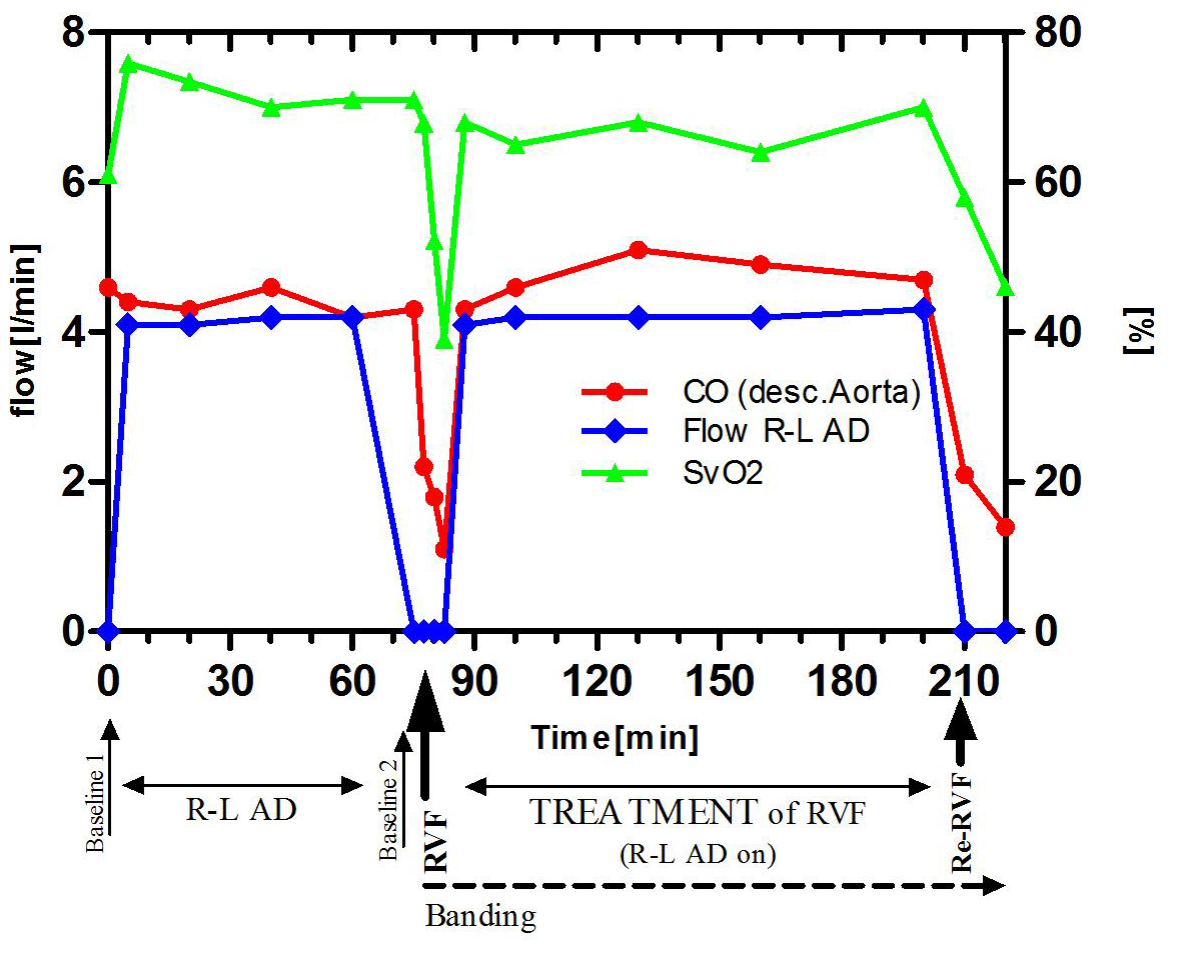
**Diagram demonstrating the relationship of the R-L AD flow with CO and SvO2 during the experiment: A complete circulatory recovery with normalization of the SvO2 is achieved**.

As fast as RVF disappeared when the R-L AD was activated, RVF returned when the system was switched off again. All parameters indicated a recurrent cardiogenic shock. Especially right sided congestion and reduced left atrial filling reoccurred with a CVP around 18 and a concordant drop of the LAP down to around 5 mmHg. [Figure 4: Diagram of the time course of MAP and RVP]; [Figure 5: Diagram of the time course of CVP and LAP]; [Figure 6: Diagram demonstrating the relationship of the R-L AD flow with CO and SvO2]

### c) Echocardiography

Baseline right ventricular systolic function (TAPSE(E 1): 20 ± 1.9 mm) and diameter values (RVd(E 1): 29 ± 3.2 mm) were within normal ranges. The heart presented good overall contractility. Within a minute of raised afterload (RVF) the right ventricle showed steady loss of systolic function and growing global dilatation, while the left ventricle appeared increasingly volume-depleted causing ventricular septal shift to the left. The right ventricular systolic function was severely reduced (TAPSE(E 2): 8 ± 1.0 mm) with severe dilatation right ventricle (RVd(E 2): 42 ± 2.1 mm). After the R-L AD was initiated the septal shift reversed quickly. Systolic function recovered gradually, but remained mildly impaired (TAPSE(E 3): 15 ± 1.7 mm). The right ventricular size returned to above normal values (RVd(E 3): 23 ± 2.5 mm), while left ventricular filling appeared normalized during pump support. Tissue doppler imaging investigations (data not shown) further confirmed a recovery from a severely injured right ventricle after turning the R-L AD on. [Additional file 3: Echocardiographic parameters]

### d) Autopsy

Neither relevant intracavitary - nor system thrombus formation was found. A gross examination of the lungs, the heart and kidneys did not reveal any signs of thrombosis or infarctation. The macroscopic inspection of abdominal cavity did not show any abnormalities.

## Discussion

Right ventricular failure often exhibits the final phases of various diseases e.g. COPD, pulmonary hypertension or ARDS [11-13]. In cardiac surgery RVF is a frequent cause for postoperative cardiogenic shock and is associated with a higher mortality than left ventricular failure [14-16]. Although great progress has been made in the field of mechanical circulatory support in general, right - sided circulatory assist is still associated with poor results [15,16]. Still some relevant issues in mechanical treatment of RVF are unclear: Neither the role of decompression of the pulmonary circulation in mechanical right ventricular assistance is defined nor its effects on remodeling mechanisms of the RV or pulmonary circulation. Furthermore there is no evidence which right ventricle (and the needed duration) can be assisted to full recovery in which underlying disease.

In this context we aimed for a new approach to assist the failing right ventricle by oxygenated bypassing of the pulmonary circulation from the right to the left atrium.

This approach, even though initially medically challenging, combines the following advantages. At first the stroke work of the right ventricle can be fully taken over by the system. Secondly, by not returning blood into the pulmonary circuit right ventricular afterload is diminished and moreover reduced transpulmonary blood-flow with decreased left sided filling (so called serial ventricular interdependence) is overcome by returning blood into the left - sided cavities. Additionally the use of a gas exchange element in the system allows optimal adjustment of oxygenation and decarboxylation, which is often crucial in RVF.

We designed such a system under the aspect, that a minimally invasive or even percutaneous implantation should be realizable in the near future. In the present study we investigated this device regarding feasibility, its efficacy in the treatment of RVF and side-effects. An at least in part open-chest implantation of the cannulas and instrumentation was chosen to simplify the investigations in a hypertension induced RVF. Our experiments proved, that the implantation of the system is easy to perform and a safe procedure without the need for cardiopulmonary bypass or extensive surgical procedures. Once the system was implanted, there was no relevant detoriation of circulatory parameters detectable neither without nor with pumping of 4-5 liters per minute. This demonstrated an overall excellent tolerance of the "Right-Left Assist Device" what seems to be important especially for weaning.

Experimentally we could demonstrate an excellent efficacy of the R-L AD to treat RVF. Activating the R-L AD led to a very fast recovery from cardiogenic shock. Whereas in the "RVF" state CVP and RVP increased, LAP and MAP decreased. With activation of the R-L AD these pressures reversed or even behaved reciprocal, what was interpreted as efficient overcoming of serial ventricular interdependence (reduced transpulmonary bloodflow to the left atrium) and also efficient reduction in right ventricular afterload. The gas exchange parameters demonstrated excellent and especially easy to alter without the necessity of aggressive ventilation. The findings of an excellent right ventricular decompression with immediate recovery of right ventricular function were also confirmed by echocardiography.

We could not detect any relevant negative side effects. There was virtually no contamination with air or gas respectively. In the post mortem examinations we could not find any signs of thrombus formation or peripheral embolism.

To our knowledge this is the first systematic investigation regarding the treatment of RVF by circumventing pulmonary circulation. Only Herlihy et al reported in 2009 an attempt in two patients to perform an ECMO support via Tandem Heart System's Catheters from the right atrium transseptal into the left atrium [17]. Although their primary aim was an improved delivery of oxygen to the brain and the avoidance of left ventricular distension by a veno-arterial ECMO (what they achieved), they concluded, that "the catheter system can provide novel and perhaps superior vascular routes for the delivery of ECMO". They did not highlight the pathophysiologic advantages of this perfusion route in RVF. In contrast we consider the approach of Wang and Zwischenberger, which introduced their concept of an "Ambulatory Oxygenator Right Ventricular Assist Device for Total Right Heart and Respiratory Support" in 2007 [18] more suited for chronic pulmonary support, because right ventricular recovery is not intended primarily. Nevertheless in 2011 Camboni published an experimental study with creation of an ASD and ECMO assistance in a model of also hypertension induced RVF [19]. He could demonstrate an overall beneficial effect of this approach, although the creation and the adjustment of various parameters like the size of the ASD and tricuspid regurgitation appeared difficult. The pathophysiologic background of this study is similar to ours, namely that shunting of oxygenated blood from the right to the left atrium avoids the overall deleterious effects of RVF. Compared to this study a much better control of all parameters like the amount of shunted volume or the degree of gasexchange seems to be possible by a R-L AD. Our approach of a thoracotomy to the left atrium was most of all chosen because it was necessary anyway for the RVF-model of pulmonary artery banding and thereby the easiest way to introduce a cannula into the left atrium. Nevertheless a non-thoractomy introduction of the cannula into the left atrium was preliminary already realized by a transvenous-transseptal approach. A matter of current investigation is a transvenous double lumen cannula which collects blood inside the right atrium and returns it transseptally into the left atrium by only one cannula inserted through the right internal jugular vein. The relative complexicity of our approach (in comparison to a delivery into the pulmonary artery) did not show any problems. The need for anticoagulation with these types of components is not really increased because the intended ACT should be 180 seconds. On the other hand this slightly increased complexicity (in comparison to conventional v-v or v-pa, but not v-a ECMO's) offers a greatly improved hemodynamic and respiratory control.

### Limitations

In this experimental study no high fidelity cardiopulmonary recording was used. The investigation of other RVF models and the determination of long term results are a matter of further investigations. Also a real comparison of our new approach with classical ECMO and RVAD seems necessary.

## Conclusion

We were able to demonstrate, that our newly developed "Right-Left Assist Device" is a feasible technique without relevant side effects in the acute setting. Operation of the system without RVF does not lead to significant circulatory changes whereas it is extremely effective in restoring normal hemodynamics in acute RVF. This approach with enhanced decompression of the right ventricle and improved left atrial filling might be further developed into a new option for the mechanical assistance of the failing right ventricle with the aim of recovery.

## List of abbreviations

ASD: atrial septal defect; CO: cardiac output; CVP: central venous pressure; Fio2: fraction of inspired Oxygen; Fr.: French (1 Fr. = 0,33 mm); Hg: mercury; ILA: interventional lung assist; LAP: left atrial pressure; MAP: mean arterial pressure; P: partial pressure of gas; Rpm: revolutions per minute; RVAD: right ventricular assist device; RVF: right ventricular failure; RVP: right ventricular pressure; SvO2: mixed venous saturation with oxygen.

## Competing interests

The authors declare that they have no competing interests.

## Authors' contributions

JS and MH.: performed experiments surgically, and drafted the manuscript; CS performed all anaesthesiologic procedures; NH performed the echocardiography; AA was responsible for data collection; CN and US were responsible for the system design and pumps; AM revised the manuscript critically for important intellectual content; RA revised the manuscript critically for important intellectual content and gave the final approval. HG and YA were responsible for the preparation of the experiments and helped all others: they are preparing their doctor thesis of this work. All authors read and approved the final manuscript.

## Supplementary Material

Additional file 1 **Experimental protocol: chronological order of measuring times**. Da = complete data acquisition, E = echocardiographic examination. Click here for file

Additional file 2 **Relevant data from the experiments according to protocol**. Click here for file

Additional file 3 **Echocardiographic parameters measured according to the protocol demonstrating right ventricular recovery by the "treatment of RVF"**. Click here for file
