# Supplementary material for: Feasibility and efficacy of bypassing the right ventricle and pulmonary circulation to treat right ventricular failure: an experimental study
Source: J Cardiothorac Surg. 2012 Feb 6;7:15. doi: 10.1186/1749-8090-7-15 (PMC3293723; doi:10.1186/1749-8090-7-15)
Supplement: Additional file 1 — Experimental protocol: chronological order of measuring times. Da = complete data acquisition, E = echocardiographic examination. [file 1749-8090-7-15-S1.DOC]

| **Section name:** | Time **(min):** | Procedure/ Measurement: |
| --- | --- | --- |
| **“Baseline I”** | 0 | After surgery: no banding, no Assist: DA,E1 |
| **“R-L AD”** | 5 | R-L AD starts running with 4,2 l/min **without** RVF: DA |
|  | 20 | DA |
|  | 40 | DA |
|  | 60 | DA |
|  | 65 | DA; then R-L AD Stop |
| **“Baseline II”** | 70 | R-L AD not running; no banding: DA |
| **“RVF”** | 77,5 | Pulmonary artery banding; R-L AD off: DA |
|  | 80 | Pulmonary artery banding; R-L AD off: DA; E2 |
|  | 82,5 | Pulmonary artery banding; R-L AD off: DA |
| **“Treatment of RVF”** | 87,5 | Pulmonary artery banding; R-L AD starts running with 4,2 l/min: DA |
|  | 100 | Pulmonary artery banding; R-L AD running with 4,2 l/min: DA, E3 |
|  | 130 | Pulmonary artery banding; R-L AD running with 4,2 l/min: DA |
|  | 160 | Pulmonary artery banding; R-L AD running with 4,2 l/min: DA |
|  | 200 | Pulmonary artery banding; R-L AD running with 4,2 l/min: DA |
| **“Re-RVF”** | 210 | Pulmonary artery banding; R-L AD turned off: DA |
|  | 220 | Pulmonary artery banding; R-L AD off: DA; End of experiment; Autopsy after euthanasia. |
