# Supplementary material for: Feasibility and efficacy of bypassing the right ventricle and pulmonary circulation to treat right ventricular failure: an experimental study
Source: J Cardiothorac Surg. 2012 Feb 6;7:15. doi: 10.1186/1749-8090-7-15 (PMC3293723; doi:10.1186/1749-8090-7-15)
Supplement: Additional file 2 — Relevant data from the experiments according to protocol. [file 1749-8090-7-15-S2.DOC]

|  | **B**aseline **I** | **“R-L AD” (no** RVF**)** | | | | Baseline **II** | RVF | | | Treatment of RVF | | | | | Re-RVF | |
| --- | --- | --- | --- | --- | --- | --- | --- | --- | --- | --- | --- | --- | --- | --- | --- | --- |
|  | | | | | | | **|-----------Pulmonary artery banding------------|** | | | | | | | | | |
| **Time**  **[min]** | **0** | **5** | **20** | **40** | **60** | **75** | **77,5** | **80** | **82,5** | **87,5** | **100** | **130** | **160** | **200** | **210** | **220** |
| **Flow**  **[l/min]** | 0 | 4,1  ±0,3 | 4,1  ±0,1 | 4,2  ±0,3 | 4,2  ±0,2 | 0 | 0 | 0 | 0 | 4,2  ±0,5 | 4,2  ±0,2 | 4,2  ±0,4 | 4,2  ±0,1 | 4,3  ±0,3 | 0 | 0 |
| **CO _desc. aorta_**  **[l/min]** | 4,6  ±1,2 | 4,4  ±1 | 4,3  ±0,9 | 4,6  ±1,2 | 4,2  ±1,4 | 4,3  ±0,8 | 3,9  ±1,3 | 1,8  ±1,7 | 1,1  ±0,7 | 4,3  ±1,6 | 4,6  ±1,9 | 5,1  ±1,5 | 4,9  ±1,0 | 4,7  ±1,4 | 2,1  ±1,2 | 1,4  ±1,6 |
| **MAP**  **[mmHg]** | 75  ±6,9 | 72  ±6,4 | 69  ±8,9 | 70  ±7,8 | 72  ±9,9 | 75  ±6,4 | 42  ±8,2 | 37  ±7,4 | 35  ±13 | 85  ±9,3 | 83  ±8,4 | 80  ±7,0 | 78  ±8,0 | 76  ±6,8 | 38  ±7,7 | 35  ±6,8 |
| **RVP _syst_**  **[mmHg]** | 34  ±6,0 | 35  ±6,9 | 31  ±7,8 | 26  ±5,7 | 30  ±6,4 | 27  ±6,3 | 48  ±6,1 | 38  ±5,8 | 40  ±9,6 | 24  ±7,4 | 27  ±5,7 | 28  ±5,1 | 24  ±5,0 | 24  ±7,3 | 42  ±6,2 | 31  ±5,8 |
| **CVP mean [mmHg]** | 8  ±4,3 | 3  ±1,6 | 4  ±2,3 | 3  ±2,2 | 4  ±2,7 | 7  ±2,2 | 15  ±5,7 | 18  ±6,2 | 19  ±3,3 | 4  ±2,4 | 3  ±1,7 | 3  ±2,8 | 4  ±1,9 | 3  ±2,0 | 18  ±2,2 | 16  ±5,3 |
| **LAP mean [mmHg]** | 9  ±2,7 | 21  ±3,3 | 22  ±2,8 | 23  ±3,6 | 19  ±3,5 | 10  ±2,1 | 4  ±2,1 | 3  ±2,4 | 5  ±2,9 | 21  ±3,3 | 22  ±3,7 | 24  ±2,9 | 21  ±2,5 | 23  ±3,1 | 7  ±2,8 | 3  ±2,4 |
| **PaO2**  **[kPa]** | 13  ±5,5 | 29,5  ±4,2 | 32,2  ±5,5 | 30,6  ±4,7 | 31,9  ±3,9 | 12,,6  ±2,4 | 14,3  ±4,7 | 12,8  ±3,6 | 10,3  ±5,3 | 27,9  ±4,1 | 34  ±2,1 | 33,4  ±3,7 | 32,5  ±1,1 | 32  ±2,9 | 9,5  ±3,2 | 7,6  ±3,6 |
| **SvO2 [%]** | 61  ±5,5 | 75,9  ±4,4 | 73,4  ±5,7 | 70  ±6,9 | 71  ±5,0 | 66  4,7 | 67,9  ±4,8 | 52  ±7,9 | 39  ±5,9 | 68  ±5,9 | 65  ±4,6 | 68  ±6,8 | 64  ±5,7 | 70  ±4,4 | 58  ±5,9 | 46  ±7,9 |
