# Supplementary material for: Feasibility and efficacy of bypassing the right ventricle and pulmonary circulation to treat right ventricular failure: an experimental study
Source: J Cardiothorac Surg. 2012 Feb 6;7:15. doi: 10.1186/1749-8090-7-15 (PMC3293723; doi:10.1186/1749-8090-7-15)
Supplement: Additional file 3 — Echocardiographic parameters measured according to the protocol demonstrating right ventricular recovery by the "treatment of RVF". [file 1749-8090-7-15-S3.DOC]

|  | E1  Baseline I | E2  “RVF”  (pump off) | E3  “Treatment of RVF”  (pump on) |
| --- | --- | --- | --- |
| RVd [mm] | 29±3.2 | 42±2.1 | 23±2.5 |
| TAPSE [mm] | 20±1.9 | 8±1.0 | 15±1.7 |
